# Supplementary material for: Mobilization of endocannabinoids by midbrain dopamine neurons is required for the encoding of reward prediction
Source: Nat Commun. 2023 Nov 20;14:7545. doi: 10.1038/s41467-023-43131-3 (PMC10662422; doi:10.1038/s41467-023-43131-3)
Supplement: Supplementary file 1 — Supplementary Information [file 41467_2023_43131_MOESM1_ESM.pdf]

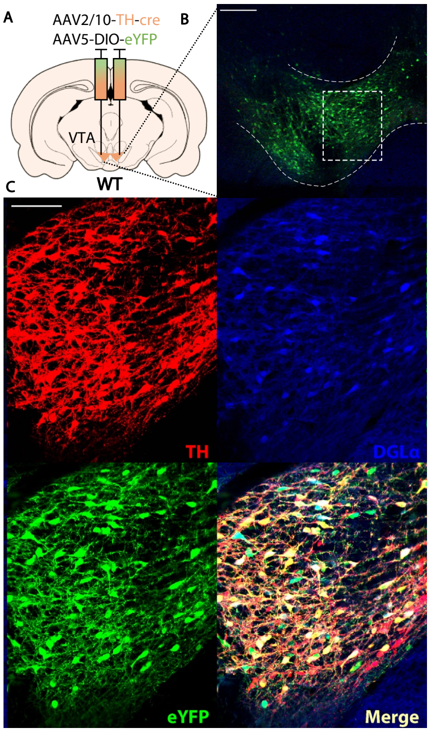

Supplementary Figure 1. ***Immunohistological verification of VTA dopamine neuron transduction.***

A) Schematic showing viral transduction of VTA dopamine neurons after bilateral injection of AAV2/10-TH-cre and AAV5-DIO-eYFP. **B)** Confocal VTA coronal section showing staining (green) for eYFP (scale bar = 200 μm). **C)** Confocal images of the VTA showing immunofluorescence for TH (red), eYFP (green) and DGLα (blue) (scale bar = 50 μm). Merged staining indicates high co-localization of eYFP+ and TH+ positive cells (n = 7 replicates).

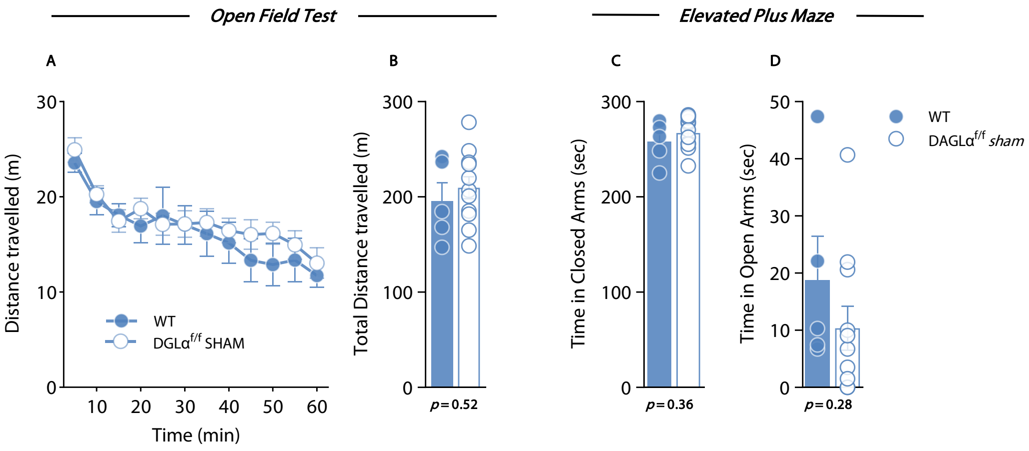

Supplementary Figure 2. A) DGLα$^{f/f}$ *sham* and WT mice exhibit similar time-course spontaneous locomotor activity in an open field test (two-sided two-way RM ANOVA; $^{genotype}F_{1,14} = 0.42$, $p = 0.52$; $^{time\ x\ genotype}F_{11,154} = 0.55$, $p = 0.86$). **B)** WT and DGLα$^{f/f}$ *sham* mice total distance traveled during the open field test (two-sided $t_{14} = 0.65$, $p = 0.52$) (WT, n = 5; DGLα$^{f/f}$ *sham,* n = 11). **C,D)** Absence of non-specific phenotypic effects in terms of anxiety-like behaviors as evidenced by similar time spent in the closed (two-sided $t_{14} = 0.92$, $p = 0.36$) and open arms (two-sided $t_{14} = 1.10$, $p = 0.28$) of an elevated plus maze test (WT, n = 5; DGLα$^{f/f}$ *sham,* n = 11). Data are presented as mean ± SEM (error bars).

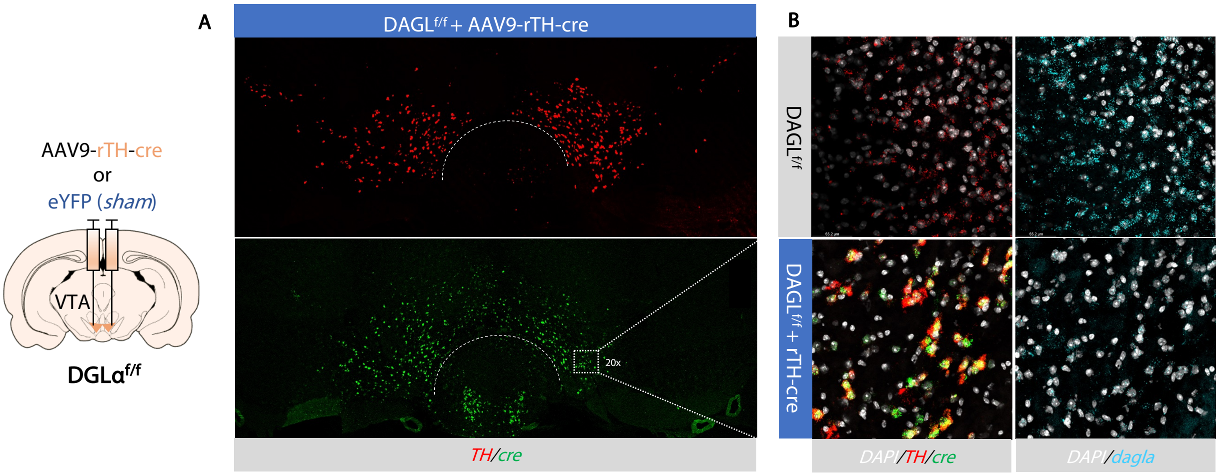

Supplementary Figure 3. ***Viral delivery of cre recombinase on TH+ cells and specific deletion of DGL mRNA transcripts in the VTA.*** **A)** 6 weeks after AAV9-rTH-cre bilateral injection in the VTA, *cre* recombinase transcripts are observed within the VTA (left, 5x magnification). **B)** Upon closer examination (right, 20x magnification), *cre* recombinase punctae (green) are highly co-localized with TH mRNA transcripts (red). Expression of *dagla* mRNA transcripts, which are also found in TH- cell types, is greatly reduced after TH^{cre}:DGLα$^{f/f}$ recombination in the VTA (n = 3 replicates).

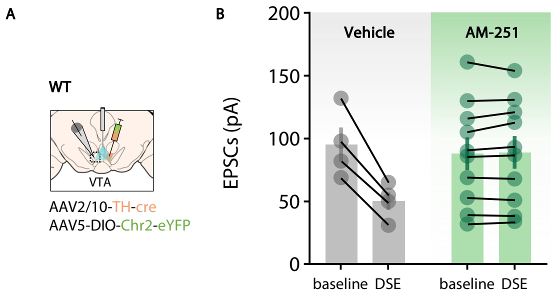

Supplementary Figure 4. ***Depolarization-induced suppression of excitation in VTA dopamine cells is governed by a CB1R-dependent mechanism.*** **A)** WT mice expressing ChR2 in VTA dopamine neurons upon recombination with *cre* sequence (driven by a TH-cre viral construct). **B)** Suppression of EPSCs following high-frequency optostimulation of dopamine neurons (DSE) is abolished by bath application of the CB1R antagonist AM251 (two-way RM ANOVA; $^{optostim}F_{1,12} = 77.81$, $p > 0.001$; $^{drug}F_{1,12} = 0.52$, $p = 0.48$; $^{optostim\ x\ drug}F_{1,12} = 84.30$, $p > 0.001$) (control, n = 4; AM251, n = 10). Data are presented as mean ± SEM (error bars).

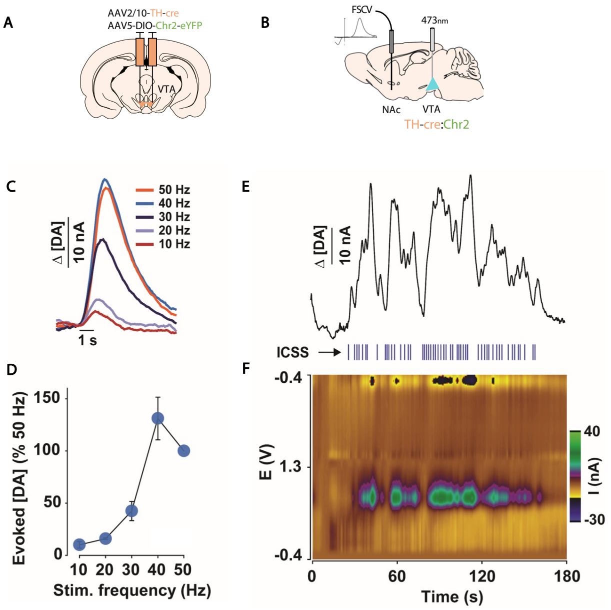

Supplementary Figure 5. Schematic representation showing viral transduction of VTA dopamine neurons (**A**), and FSCV electrode placement in the NAc as well as optical fiber placement in the midbrain (**B**). **C)** Frequency-dependent dopamine release evoked by optical stimulation in awake WT mice (n = 3). **D)** Mean dopamine concentration change (Δ[DA]) during each stimulation and mean maximal Δ[DA] relative to the 50 Hz stimulation (n = 3). Data are presented as mean ± SEM (error bars). **E)** Representative NAc FCSV recording (180 s) following oICSS of VTA dopamine neurons (n = 3 replicates). Blue lines indicate self-stimulation timestamps. Behaviorally-locked NAc phasic dopamine transients illustrate effective targeting of VTA dopamine neurons by the TH-cre construct (AAV2/10-TH-cre). **F)** Color plot displaying background-subtracted cyclic voltammograms sequentially in time (n = 3 replicates).

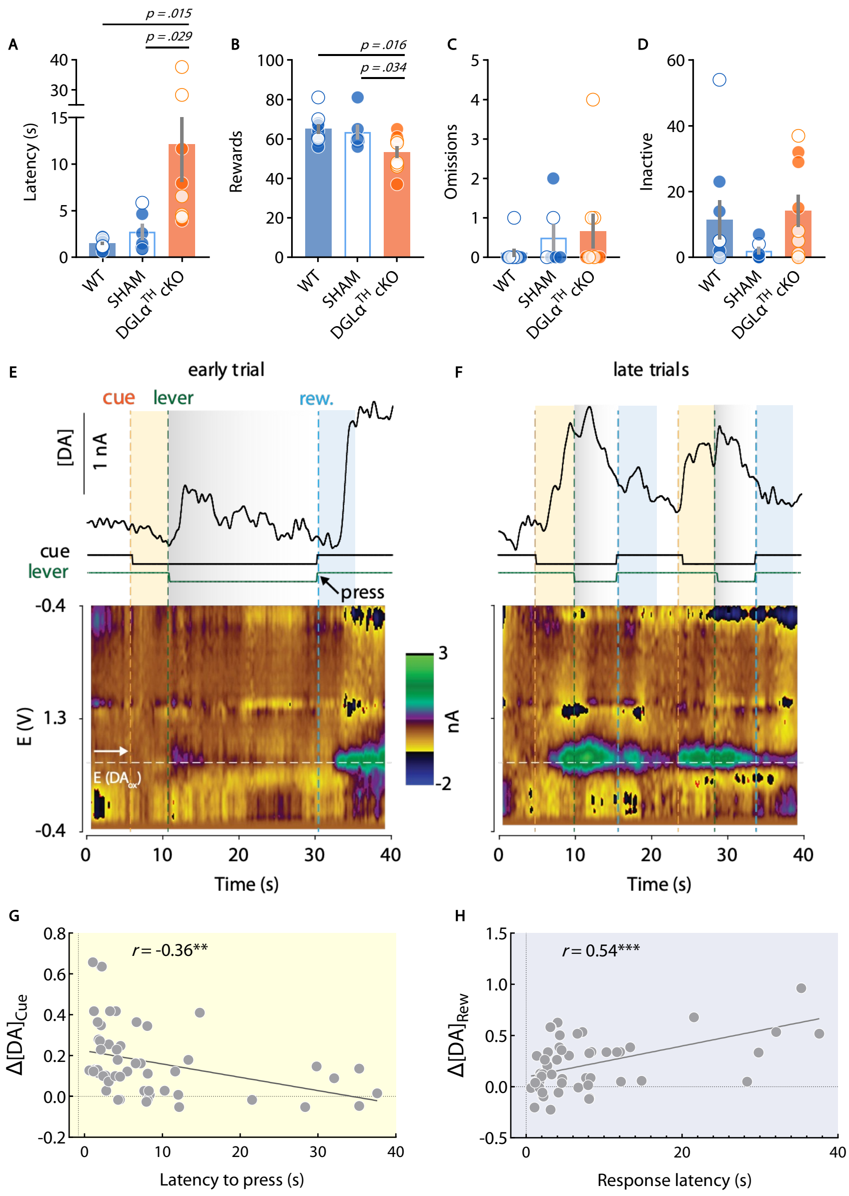

Supplementary Figure 6. ***Impaired cue-directed behavior after DGLa deletion from VTA dopamine neurons.*** **A)** Cue-evoked conditioned responding latency during VTO testing is impaired in DGLαTH cKO compared to both WT and DGLα$^{f/f}$ *sham* mice (two-sided one-way ANOVA; $F_{2,21}$ = 5.01, p = 0.01 Holm-Sidak *post hoc* tests shown in panel). **B)** Number of rewards obtained during variable time-out (VTO) testing (two-sided one-way ANOVA; $F_{2,21}$ = 4.79, p = 0.01, Holm-Sidak *post hoc* tests shown in panel). **C)** Omission errors (latency to press > 60 s) during VTO testing (two-sided one-way ANOVA; $F_{2,21}$ = 0.47, p = 0.62). **D)** Number of VTO inactive lever presses (two-sided one-way ANOVA; $F_{2,21}$ = 1.64, p = 0.21) [WT, n = 9 (5M, 4F); DGLα$^{f/f}$ sham = 6 (4M, 2F); DGLαTH cKO = 9 (4M, 5F)]. **E,F)** Representative FSCV recordings of dopamine release dynamics for a representative trial during (**E**) early or (**F**) late VTO training. (*Upper panels*) Distal cue (yellow), lever extension (proximal cue, green) and reward delivery (blue) are indicated by colored dotted lines. Time intervals used for quantification analyses are indicated by the respective colored shaded areas (distal cue and reward delivery: 5 s; Lever extension: variable interval comprehending from lever extension to reward delivery). (*Lower panels*) Two- dimensional color representation of cyclic voltammetric data. Changes in current at the carbon-fiber electrode are indicated in color. **G)** Greater cue-evoked NAc dopamine release predicts shorter conditioned response latencies during VTO (two-sided Pearson's r = -0.36, p = 0.007). **H)** Conversely, reward-evoked NAc dopamine release positively correlates with longer response latency (two-sided Pearson's r = 0.54, p = 2·10^{-4}).
