# Supplementary material for: Mobilization of endocannabinoids by midbrain dopamine neurons is required for the encoding of reward prediction
Source: Nat Commun. 2023 Nov 20;14:7545. doi: 10.1038/s41467-023-43131-3 (PMC10662422; doi:10.1038/s41467-023-43131-3)
Supplement: Supplementary file 3 — Reporting Summary [file 41467_2023_43131_MOESM3_ESM.pdf]

nature portfolio

Corresponding author(s): Joseph F. Cheer

Last updated by author(s): Oct 10, 2023

Reporting Summary

Nature Portfolio wishes to improve the reproducibility of the work that we publish. This form provides structure for consistency and transparency in reporting. For further information on Nature Portfolio policies, see our Editorial Policies and the Editorial Policy Checklist.

Statistics

For all statistical analyses, confirm that the following items are present in the figure legend, table legend, main text, or Methods section.

n/a	Confirmed	
☐	☒	The exact sample size (*n*) for each experimental group/condition, given as a discrete number and unit of measurement
☐	☒	A statement on whether measurements were taken from distinct samples or whether the same sample was measured repeatedly
☐	☒	The statistical test(s) used AND whether they are one- or two-sided<br>*Only common tests should be described solely by name; describe more complex techniques in the Methods section.*
☐	☒	A description of all covariates tested
☐	☒	A description of any assumptions or corrections, such as tests of normality and adjustment for multiple comparisons
☐	☒	A full description of the statistical parameters including central tendency (e.g. means) or other basic estimates (e.g. regression coefficient) AND variation (e.g. standard deviation) or associated estimates of uncertainty (e.g. confidence intervals)
☐	☒	For null hypothesis testing, the test statistic (e.g. *F*, *t*, *r*) with confidence intervals, effect sizes, degrees of freedom and *P* value noted<br>*Give P values as exact values whenever suitable.*
☒	☐	For Bayesian analysis, information on the choice of priors and Markov chain Monte Carlo settings
☒	☐	For hierarchical and complex designs, identification of the appropriate level for tests and full reporting of outcomes
☒	☐	Estimates of effect sizes (e.g. Cohen's *d*, Pearson's *r*), indicating how they were calculated

Our web collection on statistics for biologists contains articles on many of the points above.

Software and code

Policy information about availability of computer code

Data collection	Med-PC IV, EthovisionXT 17, LabView 2020
Data analysis	Matlab 2020a, GraphPad Prism 9.5, SigmaPlot 14.5, Demon Analysis Software

For manuscripts utilizing custom algorithms or software that are central to the research but not yet described in published literature, software must be made available to editors and reviewers. We strongly encourage code deposition in a community repository (e.g. GitHub). See the Nature Portfolio guidelines for submitting code & software for further information.

Data

Policy information about availability of data

All manuscripts must include a data availability statement. This statement should provide the following information, where applicable:

- Accession codes, unique identifiers, or web links for publicly available datasets
- A description of any restrictions on data availability
- For clinical datasets or third party data, please ensure that the statement adheres to our policy

The data that support the findings of this study have been deposited in a Figshare repository under accession code ...

Research involving human participants, their data, or biological material

Policy information about studies with human participants or human data. See also policy information about sex, gender (identity/presentation), and sexual orientation and race, ethnicity and racism.

Reporting on sex and gender	N/A
Reporting on race, ethnicity, or other socially relevant groupings	N/A
Population characteristics	N/A
Recruitment	N/A
Ethics oversight	N/A

Note that full information on the approval of the study protocol must also be provided in the manuscript.

Field-specific reporting

Please select the one below that is the best fit for your research. If you are not sure, read the appropriate sections before making your selection.

☒ Life sciences ☐ Behavioural & social sciences ☐ Ecological, evolutionary & environmental sciences

For a reference copy of the document with all sections, see nature.com/documents/nr-reporting-summary-flat.pdf

Life sciences study design

All studies must disclose on these points even when the disclosure is negative.

Sample size	No sample-size calculation was performed. Sample sizes were chosen based on previous studies demonstrating behavioral effects following similar manipulations with comparable sample sizes (Kondev et al., 2023, PMID:37703881; Marcus et al., 2020, PMID:31948734).
Data exclusions	No data was excluded.
Replication	All behavioral findings (Fig1f-h, Fig2, Fig3l-o, Fig4c) were replicated at least once. All histology (Fig1d, Fig3h) was replicated in minimally 3 animals and representatives images were chosen for publication. No reproducibility problems found.
Randomization	Allocation of subjects to each of the experimental groups (sham/cKO) was random.
Blinding	Once surgery procedures were completed, animal IDs were coded to ensure that experimenters were blinded to the subject"s group.

Reporting for specific materials, systems and methods

We require information from authors about some types of materials, experimental systems and methods used in many studies. Here, indicate whether each material, system or method listed is relevant to your study. If you are not sure if a list item applies to your research, read the appropriate section before selecting a response.

Materials & experimental systems

n/a	Involved in the study	
☐	☒	Antibodies
☒	☐	Eukaryotic cell lines
☒	☐	Palaeontology and archaeology
☐	☒	Animals and other organisms
☒	☐	Clinical data
☒	☐	Dual use research of concern
☒	☐	Plants

Methods

n/a	Involved in the study	
☒	☐	ChIP-seq
☒	☐	Flow cytometry
☒	☐	MRI-based neuroimaging

Antibodies

Antibodies used	mouse anti-TH (ImmunoStar #22941; RRID: AB_572268), rabbit anti-DGLa (ThermoFisher #115090, RRID: AB_2899726), donkey anti-mouse Alexa 647 (Jackson #715605151, RRID: AB_2340863) and donkey anti-rabbit Alexa 488 (Jackson #711545152, RRID: AB_2313584).

Validation	Anti-TH(ImmunoStar #22941): https://www.immunostar.com/wp-content/uploads/22941-1942001.pdf<br><br>Anti-DGLa (Jackson #711545152): https://www.jacksonimmuno.com/catalog/products/711-545-152

Animals and other research organisms

Policy information about studies involving animals; ARRIVE guidelines recommended for reporting animal research, and Sex and Gender in Research

Laboratory animals	Male and female C57BL/6 (WT and DGLaf/f) mice (3-6 mo) were used in the study. Mice were housed at 24 degrees Celsius with a humidity between 40-50%.
Wild animals	The study did not involve wild animals.
Reporting on sex	Sex as a biological variable (SABV) was considered in the study design. All behavioral experiments included an equal amount of male and female subjects. The portion of male and female subjects per group is provided on each figure legend. In the figure panels, open circles represent data from female subjects and closed circles from male animals. For statistical purposes, sex was first added as an additional factor in all the analyses performed. When no sex effect was detected, males and females were collapsed in the same group and analyses proceed without including SABV.
Field-collected samples	The study did not involve samples collected from the field.
Ethics oversight	Animal care and experimental procedures conformed to the National Institutes of Health Guide for the Care and Use of Laboratory Animals and were approved by the Institutional Animal Use and Care Committee at the University of Maryland, Baltimore.

Note that full information on the approval of the study protocol must also be provided in the manuscript.

Plants

Seed stocks	N/A
Novel plant genotypes	N/A
Authentication	N/A
